# The adoption of a new diagnostic technology for tuberculosis in two Brazilian cities from the perspective of patients and healthcare workers: a qualitative study

**DOI:** 10.1186/s12913-015-0941-x

**Published:** 2015-07-21

**Authors:** Kenneth R. de Camargo, Carla R. Guedes, Rosângela Caetano, Alexandre Menezes, Anete Trajman

**Affiliations:** Departamento de Planejamento e Administração em Saúde, Instituto de Medicina Social, Universidade do Estado do Rio de Janeiro, (R. S. Fco. Xavier, 524 7º Andar Bloco D.), Rio de Janeiro, RJ (20559-900) Brazil; Departamento de Saúde e Sociedade, Instituto de Saúde Coletiva, Universidade Federal Fluminense, (Rua Marquês do Paraná, 303 - 3º andar), Niterói, RJ (24030-210) Brazil; Global Health Strategies Brazil, (Largo do Machado, 21 sala 518), Rio de Janeiro, RJ (22221-020) Brazil; Internal Medicine Post-graduation Program, University Hospital, Federal University of Rio de Janeiro, (Av. Brigadeiro Trompowski s/no, Ilha do Fundão), Rio de Janeiro, RJ (21949-90) Brazil

**Keywords:** Tuberculosis, Diagnosis, Qualitative research

## Abstract

**Background:**

This article presents the qualitative component linked to a larger study of implementation of the Xpert™ MTB/Rif technology in two Brazilian cities. Despite intrinsic advantages of new health technologies, its introduction can be disruptive to existing routines, and it is thus important to understand how these innovations are perceived by the different groups involved in its regular use.

**Methods:**

This study was based on semi-structured interviews with patients, lab technicians, health care workers and managers involved with diagnosis and care of Tuberculosis (TB). The interviews had their content analyzed in order to abstract the different perspectives for the various actors.

**Results:**

For patients the changes were not perceived as significant, since their greatest concerns were related to treatment and the stigma associated with TB. The professionals in general welcomed the new technique, which dramatically decreases the workload, time and reliability of diagnosis, in their view. However, we noted difficulties with the concomitant implementation of new IT technology for recording and reporting test results, which negatively impacted the time necessary to get lab diagnosis to physicians.

**Conclusions:**

Through this analysis we detected some bottlenecks in the surrounding environment, not necessarily linked to the technology itself but which could hamper considerably its advantages.

## Background

Tuberculosis (TB) is a curable disease with high treatment success rates. Nevertheless, it figures among the top ten causes of death worldwide, with 9.4 million new cases and 1.68 million deaths in 2009, 380,000 of which occurred in HIV-positive individuals. The emergence of drug-resistant forms of TB, particular to rifampin, a highly effective drug used worldwide, and their rapid spread in Asian, African and East European countries are a particular concern [1].

Early detection of both the sensitive and resistant forms of the disease reduces loss in quality of life, morbidity, deaths and prevents the transmission of drug sensitive and resistant TB. However, a major obstacle in the control of TB is the delay in diagnosis. The provision of diagnostic services is inadequate due to the organization and infrastructure of the health services network, and to limitations of the technologies currently used, such as sputum smear and culture for *Mycobacterium tuberculosis* (MTB). The sensitivity of the former is low (especially among patients with HIV co-infection and children) and requires two patient visits to the clinic to provide the material: at least two samples are required to attain optimal, yet very low, sensitivity (around 70 %). Sensitivity of smears is even lower in paucibacillary forms of pulmonary disease, as in children and in patients infected with TB and HI. It should be noted R. de Camargothat sputum smear is a fully manual, labor-intensive process whose quality is entirely dependent on technicians’ expertise, and fluctuations in the processing influence heavily the test’s diagnostic properties. Sensitivity of culture is over 90 %, but long delays in providing results reduce its applicability for rapid medical decision and infrastructure bottlenecks hamper its wide availability in developing countries [2].

Brazil is among the 22 countries with the highest TB burden. The estimate by World Health Organization (WHO) for 2011 was 91,000 new cases and 4,900 deaths in the country [1]. Th diagnosis of pulmonary TB in Brazil relies almost exclusively on sputum smears, offered free of charge by the national health care system (Sistema Único de Saúde, or SUS). The National TB Control Program (Programa Nacional de Controle da Tuberculose – PNCT) recommends the use of culture (and drug sensitivity tests if appropriate) for diagnosing TB when specimens are difficult to obtain, in cases with a high probability of resistance and in those with usual negative smears [3]. In practice, Brazil has more than one third of new TB cases reported without bacteriological confirmation. It is estimated that 20 % of them do not represent true cases of TB, which means delay in correct diagnosis and unnecessary exposure to the risk of liver toxicity and other adverse effects from tuberculostatics [4, 5]. On the other hand, the WHO estimates that only 79 % of new and relapsed cases are detected in Brazil [1], leaving a considerable number of patients without proper care and hindering the control of the disease in the country.

After a century of virtual stagnation, new technologies for the detection of MTB are being developed, mostly based on molecular methods. One of these new technologies is the automated polymerase chain reaction-based test Xpert™ MTB/Rif, for the rapid detection of MTB and rifampicin resistance. The new test has been considered a milestone for the global control of TB, because of its high sensitivity and specificity both for detecting MTB and resistance to rifampin [2], as well as its ability to deliver results in less than two hours. In December 2010, the WHO recommended the adoption of the Xpert™ MTB/Rif [6]. The Brazilian Ministry of Health approved the incorporation of this method in March 2013, after an implementation pilot study in two high TB-incidence cities, Manaus and Rio de Janeiro.

**Fig 1:**
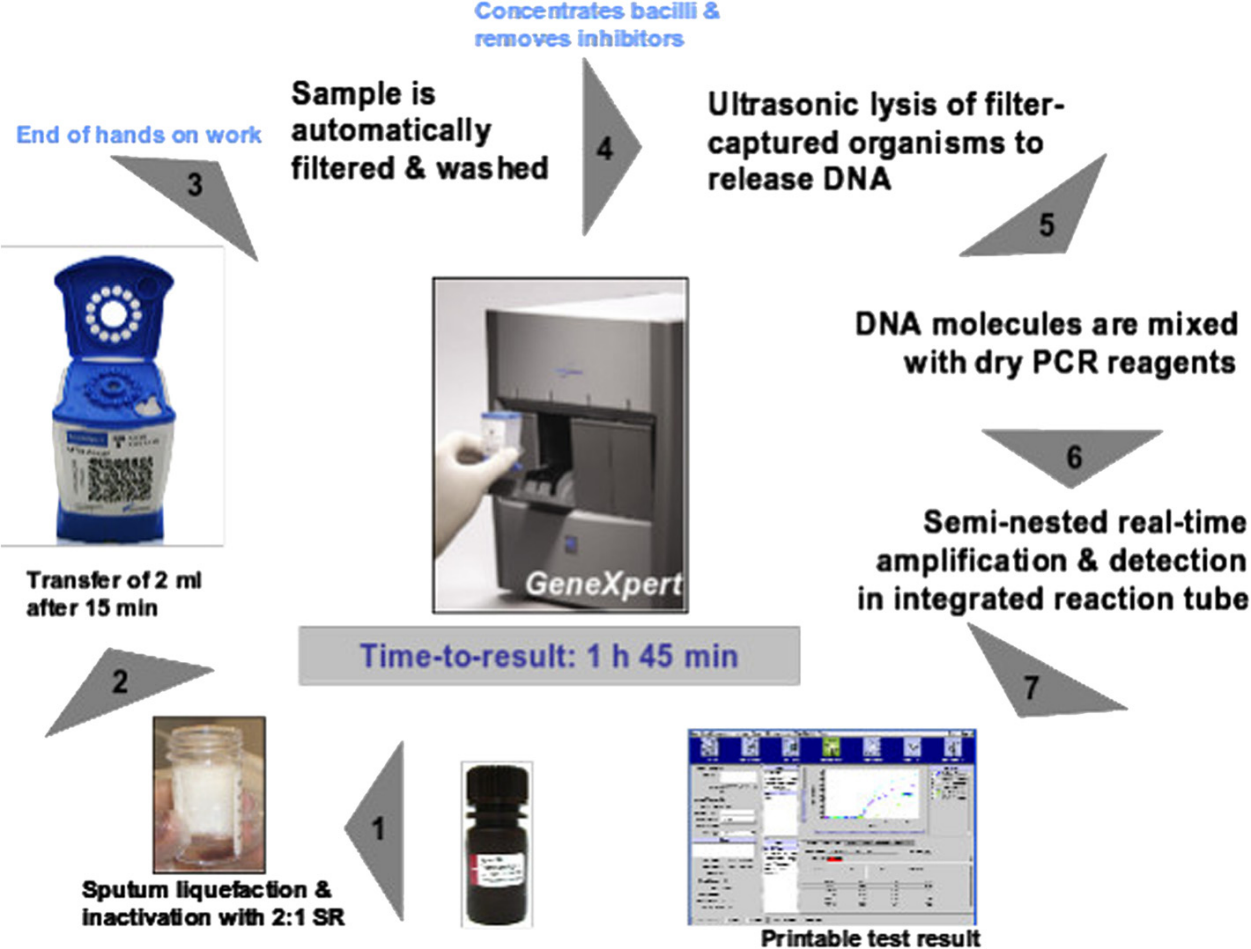
Operating cycle of the GeneXpert™ MTB/Rif equipment

The GeneXpert ™ MTB/Rif test performed on the GeneXpert ™ MTB/Rif system is a rapid molecular test for the detection of MTB and rifampicin resistance. It consists of a GeneXpert instrument, personal computer and net disposable cartridges. The system combines sample preparation in cartridges and the amplification and detection of nucleic acid DN in a fully integrated and automated instrument for analysis (Fig. 1).

The test is based on the polymerase chain reaction (PCR). A number of molecular beacons are used simultaneously to detect the presence of MTB and to diagnose rifampicin resistance as a surrogate marker for multidrug resistant disease. Species-specific primers allow the amplification of the rpoB central region of MTB DNA. Nested PCR is used to increase the sensitivity of the assay. The manual pre-treatment of the sample for the test comprises the following steps: the technician adds the buffer to sputum samples and a defined volume of this mixture is transferred to the sample chamber of the cartridge, which is then inserted into the instrument. From this point, all steps are automated: the GeneXpert ™ first catches the MTB organisms from the sputum sample in a filtering membrane. The inhibitors are then washed away from the captured cells with the buffer solution. Finally, captured cells are washed and lysed with ultrasonic energy and the released DNA is eluted through the filter membrane. The DNA solution is finally mixed with dried PCR reagents and transferred to the PCR tube for PCR and real-time detection. The cartridges are disposable and contain: i) chambers to maintain the samples and reagents; ii) a valve body comprising a piston and syringe; iii) a rotary valve system to control the movement of fluids between chambers; iv) an area for cell capturing, concentrating, washing and lysis; v) lyophilized reagents of real time PCR buffer and wash solutions; and vi) An integrated PCR reaction tube which is filled automatically by the instrument. The total processing time is 1 h and 45 min.

There has been a recent increase in the literature on innovations in health [7–10], which points out that the process of diffusion of new technologies within health care systems is complex and determined by multiple factors that can hamper or facilitate their adoption. Innovations in health – new technologies, new services and/or processes – are usually implemented with the goal of having better outcomes, more managerial effectiveness, increase cost-effectiveness or users’ experience, or any combination of those. Despite any putative advantages, however, the introduction of new technologies can be disruptive of established professional roles and processes, especially in the case of TB in which diagnostic routines have remained unchanged for decades. This can elicit various forms of resistance from the different actors involved in the process, and if that does happen, the new technology will operate at a sub-optimal level (if at all), without granting its full benefits to the various professionals and patients depending on it [11–13].

A better understanding of the elements that can influence and shape the adoption of this new technology will help the effort to introduce the test in the clinical practice and health care facilities in the country, and, consequently, contribute to the control of the disease. The goal of this study is, thus, to qualitatively evaluate the repercussions of the adoption of the Xpert™ MTB/Rif in the Brazilian Health System from the perspective of patients, health professionals and managers, considering aspects such as understanding, perception and meaning that usually are not easily amenable to quantitative approaches.

## Methods

The present study was a component of a larger implementation study, and the sites for the collection of empirical data were healthcare facilities in two Brazilian cities with high burdens of TB (Rio de Janeiro and Manaus), where the implementation study was carried out. We chose two of the ten selected areas in Rio de Janeiro, and an additional site in Manaus, according to criteria of accessibility and implementation of the new technology. The pilot implementation study was conducted in labs serving the primary care network covering the vast majority of new TB cases in both cities. The focus on primary care facilities was determined by the NTCP and local health authorities, considering the eventual applicability of this technology in the Brazilian context. In the city of Rio de Janeiro, the field sites for the qualitative research were a family clinic and a polyclinic. In Manaus, the chosen site was a polyclinic, which serves as a reference center for TB care in the city.

The qualitative component encompassed three perspectives: patients, healthcare professionals and managers.

### The patients’ perspective

Two sets of interviews were conducted with patients referred for diagnostic procedures for TB, the first set with patients whose diagnoses were based on smears and the others with patients diagnosed after the introduction of the Xpert™ MTB/Rif technology. Patients were interviewed after a visit to the TB clinic.

We used a variation of the traditional in-depth interview, a semi-structured interview; in those, the interviewer has in mind a set of topics that (s)he intends to be addressed by the interviewee, but they are not formulated as questions, and do not have a rigid sequence. As the conversation flows, the interviewer will attempt, with minimal disruption, to cover the topics [14]. The interview is a process of producing a shared narrative between interviewer and interviewee [15], and caution must be taken not to induce responses that would cater to what they suppose interviewers might be interested in hearing. Thus, we selected trained interviewers with previous experience in this methodology, which were instructed to indirectly approach the issues we wanted to address. Three interviewers conducted the interviews, under the direct supervision of one of the authors throughout the fieldwork.

The interviews were recorded and transcribed. There was no preset number of respondents, the necessary number of interviews was established by the saturation criterion, “when new data appear to add little to the understanding of the phenomenon, at least in terms of how it applies to the material investigated” [16] p65. Thus, no other patients were recruited when new interviews ceased to add new information about what was being sought in the study.

In Rio de Janeiro, eleven patients diagnosed with smears (6 men and 5 women with ages ranging 20–58 years) and nine diagnosed with Xpert™ MTB/Rif (5 men and 4 women with ages ranging 16–59 years) were interviewed. In Manaus, ten interviews were conducted with patients diagnosed with the new technology (5 men and 4 women with ages ranging 19–85 years). We did not interview patients diagnosed with smears in Manaus, since at the time of the fieldwork the new technology was fully implemented in the clinic.

The following outline of issues were addressed:

- Description of the steps to get to the clinic, identifying hurdles (queuing, scheduling, difficulties leaving the workplace);
- Views on TB and its consequences;
- Expectations regarding the diagnostic process;
- Assessment of how the diagnostic process was handled to this point;
- Main concerns and worries.

### The healthcare professionals’ perspective

We used a modified version of a technique developed by Merhy and Franco [17], the diagnostic flowchart, a specialized form of group interview. This technique is akin to a focus group, but the task that the group is required to discuss, in the original formulation, is the trajectory of patients within a specific health care service; in our case, we adapted this to discuss the trajectory of test samples and results. It should be noted that although the explicit objective of the group is to produce a flowchart, the actual dynamics of their interaction is what is really relevant. The process of discussion often results in bringing to the forefront all sorts of issues that are usually dealt without reflection, that are taken for granted, but may have great importance in the daily operation. All professionals involved in the process were gathered and they were invited to draw up, with the help of a facilitator, a flowchart that describes the steps the patient takes once it enters the unit, tracing them all the way to the end of the diagnostic process. In this case, it was adapted to include the intralaboratorial steps, retracing all the way down to the formulation of a final laboratorial diagnosis.

Two flowcharts were traced on each site, one regarding workflow before and one after the introduction of the new technique. During the preparation of the flowchart, the conversation with the team was also recorded, and this material was analyzed in the same way as the interviews.

At the Rio de Janeiro site, a physician, a nurse, a laboratory technician and an administrative staff member were involved in the preparation of the flowcharts. In Manaus, the director of the facility, one municipal health official, a physician, two nurses, a receptionist and a lab technician participated. The three field researchers participated in all the group meetings to elaborate the flowcharts.

### The managers’ perspective

We interviewed key informants at the research sites and higher-ranking positions of local health departments. They were interviewed after the introduction of the new technology.

The interviews were focused on the evaluation of the respondents about what has changed, what would be, in their opinion, the pros and cons of the new process and how they viewed the prospect of its expansion within the SUS. In Rio de Janeiro, the interviews were conducted with officers in two senior positions at the city’s health department, two laboratory directors and two officers of the facilities that served as research sites. In Manaus, three managers were interviewed: the director of the Polyclinic, a city health officer and a state health officer.

All transcripts were subjected to a content analysis [18] as a means to reduce and structure a large unstructured textual body. The text corpus was read looking for recurrences within the text and developing coding categories that could be applied to similar segments of text. Text segments coded in the same way produced synthetic aggregates and summaries were produced from such groups.

The flowcharts were compared, with emphasis on a before/after analysis, seeking to identify the changes produced by the introduction of the new technology in the laboratory workflow. The recorded material added an evaluative dimension to these changes, providing information on how the professionals see such changes.

The study was approved by the National (CONEP, #494/2011) and local Ethical Boards (CEP SMS # 236/11 and CEP FMT/HVD, no number, dated November 24, 2011). All participants signed an informed consent. None of the subjects selected for the interviews refused to participate in the study, although this option was clearly presented as available in the terms of the informed consent. To protect the identity of respondents, we used fictitious names for patients; managers were identified by numbers.

## Results

The collected data and its analysis provided a rich overview of the impact of introducing this new diagnostic approach in the settings of the study. We only report the most relevant aspects of our findings.

### The patients’s perspective

The codes used in the content analysis of the interviews were as follows:

- access to the facility: descriptions of how they got to the health facilities, difficulties and obstacles encountered;
- access within the facility: narratives about the reception and stay within the health facilities;
- trajectory within the health care system: general information on their experience with the health care system related to current diagnosis until the possibility of TB was first raised;
- diagnostic communication : how the diagnosis was communicated, who communicated and reactions to it;
- prior knowledge: information and (mis) conceptions about tuberculosis prior to getting the diagnosis;
- stigma and discrimination: narratives about situations of perceived discrimination, cultural representations of stigma, local and family lore about the disease;
- concerns: main worries related to the disease

The main concerns presented by the patients were related to the treatment (because of its long duration and some of the side effects of the drugs) and the persistent stigma attached to TB, as illustrated by interview excerpts below. What kind of technology was used for diagnostic was not their concern, and this issue was not highlighted by interviewees. Knowledge about different aspects of the disease was scarce among the interviewees, even though they all already had been diagnosed, and there were indications that they knew even less prior to diagnosis.

Access to the health facility was not an issue for most patients in all sites, although in Manaus, due to diagnostic activities being concentrated at the site of the fieldwork, patients residing in areas distant from the city center had to face long trips by bus. Most of the patients were on a medical leave due to the disease, and had their bus tickets subsidized, so the additional time was not an issue, although it could become one in case any of these conditions change.

The interviews reveal a strong stigma associated with the disease. Interviewees often reported that they omitted the diagnostic from their friends, neighbors and work colleagues for fear of social exclusion:

“In the street where I live, if people know that [I have TB], my dear, we would become celebrities. Everyone would talk [about that]! There would be so much gossiping that no one would come near [us]!” (Lucia, Rio)

“I would rather have another type of disease, that were not transmitted, because just from hearing about it, people walk away!” (Marta, Rio)

“I didn’t tell anyone, the only people who know are the folks at home. In the company I work, I’m on leave, I told it was my backbone.” (Luiz, Manaus)

“I prefer to stay away from people, so that no one will laugh [at me].” (Fatima, Manaus)

Other accounts mention a link of the diagnosis to being dirty and impure, or shameful.

“I didn’t want to tell anyone because it felt as if I was dirty. I thought it was a dirty disease. Like…, an ugly thing.” (Joyce, Ri)

“First it’s a sensation of, like, shame, that you feel” (Maíra, Manaus)

### The healthcare professionals’ perspective

#### Analysis of the flowcharts

**Fig 2:**
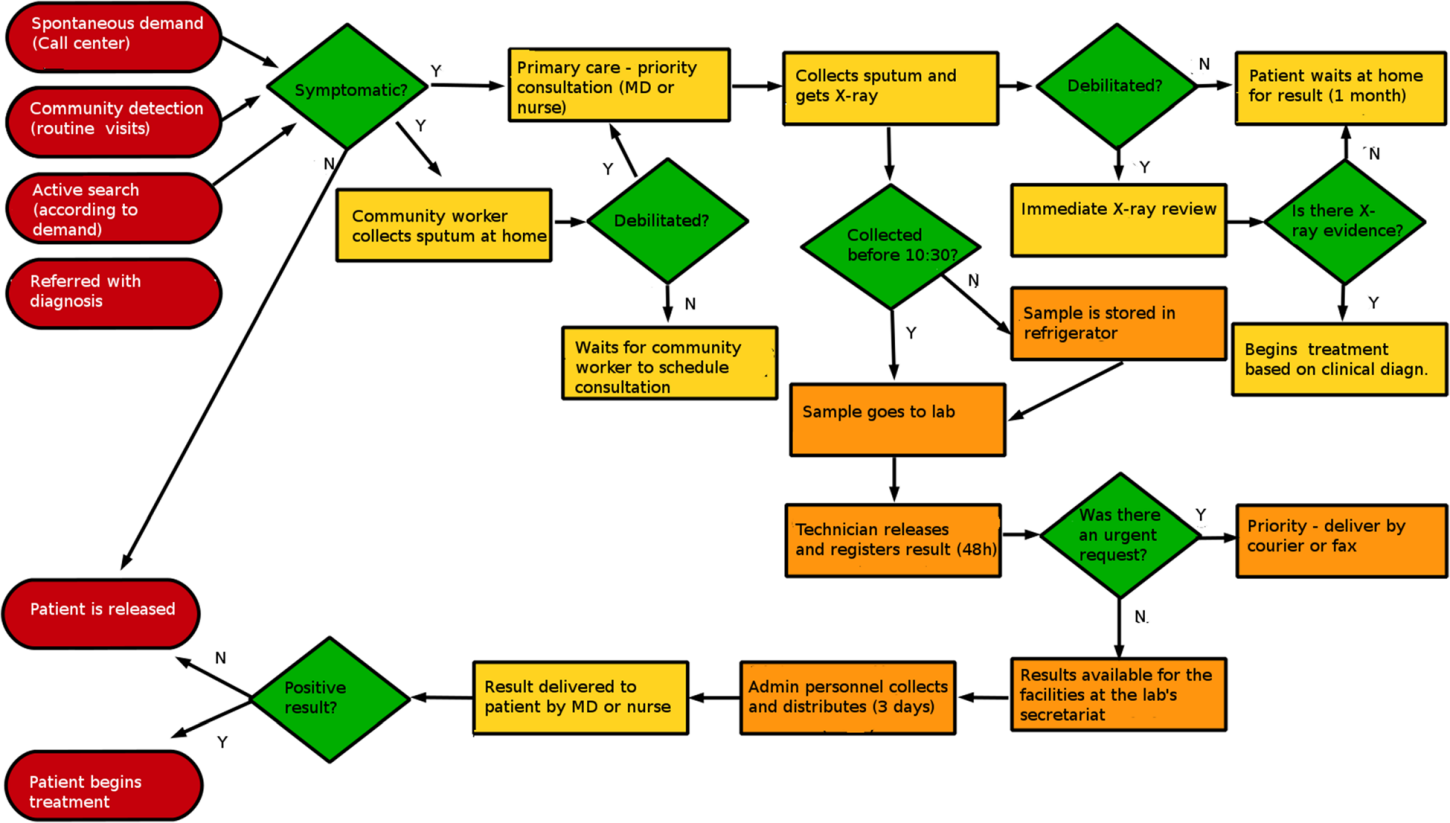
Flowchart of the old technology

**Fig 3:**
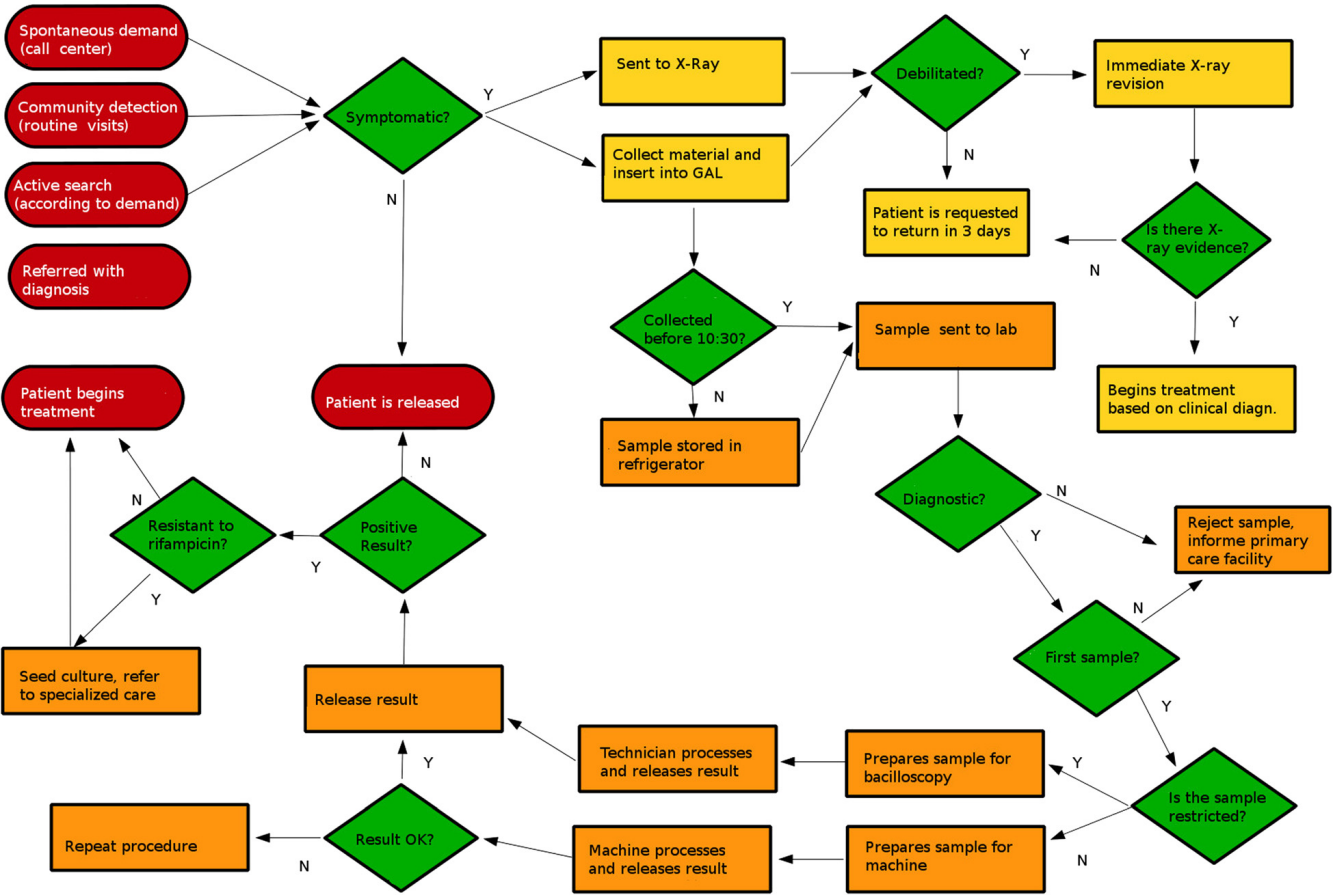
Flowchart of the new technology

We found two considerably different situations in Rio de Janeiro and Manaus, much possibly related to the more recent (and somewhat incomplete, at the time of the field work) implementation of the new technology in the former city. Figures 2 and 3 show the flowcharts for the old and new technologies in Rio, respectively, as an illustration of the flowcharts produced.

Two issues stood out in the implementation of the new technology at the Rio de Janeiro site: the changes in workflow in the laboratory and the concomitant deployment of the laboratory information system, known by the acronym GAL, whereas those steps were not concomitant in Manaus.

With the previously current technology (smears), the lab technician is in charge of all stages of the procedure: sample preparation and analysis, and data entering in the internal information system. The introduction of the Xpert™ MTB/Rif introduces a new element, a decision process that did not previously exist. The technicians need to analyze the quality of the sample (not all samples are adequate for the Xpert™ MTB/Rif processing), and depending on that analysis different procedures will be adopted. They work with two methods concomitantly: the Xpert™ MTB/Rif and smears (in the case of inadequate or insufficient samples for Xpert™ MTB/Rif processing and when the Xpert™ MTB/Rif yielded positive results, a procedure used in the pilot study protocol happening at the time of the fieldwork). Consider the exchange below between a lab technician (T) and one of the field researchers (R1) durign the elaboration of the flowchart in Rio:

“T: If yes, then we have to know if the sample is adequate for analysis. I do not know if you will write this, but you have to know if it has enough volume …

R1: Okay, so, another question: is there enough volume? Before you did not do that?

T: You must have a minimum volume to make the smear, but is much smaller than for the Gene[XPert].

R1: Do you have enough volume? Then what?

T: Then, if so, what does the sample look like? If there is some food residue, you can’t use it to do it with the Gene[XPert]. If there are hemoptoics, OK, with blood residue, you can’t use the Gene[XPert] either. So there are two constraints like that …

R1: So I’ll change the question: Are there restrictions?

T: If there is food residue, if there is blood residue, and volume less than 1 ml… (…)

T: It’s a change. So that created a moment of decision that did not exist.

R1: Which is important…

T: This was created, a moment of decision that did not exist. Which method I will use? I’m going to do the smear or what I’ll do is… the GeneXpert?

R1: Is it the lab technician that decides it on the fly?

T: Yeah, it’s the technician who decides. Did it generate more work? Because you’re working with two methods instead of one.”

Additionally, the studied site chose to assign to a member of the administrative staff the task of entering data in the GAL system.

There was no noticeable resistance or difficulty posed by the lab technicians to the new technology. They referred to it just as a “change in the works”. One possible explanation for this acceptance is the fact that the lab technicians have not lost their relevance in the workflow. They are charged with deciding which technology is the most appropriate for the analysis of the sample, in the case of the Xpert MTB/Rif they prepare samples and operate the instrument, and with the smears they still perform the usual procedure. In addition to that, they are still in charge of the internal registration procedures and of authorizing the delivery of the diagnostic results.

Regarding the time the procedure takes in the laboratory, the technicians claim that there are no significant differences.

According to their discussion, the difference in time is mostly in reference to how quickly results are delivered. The rollout of the GAL, despite being yet a new technology under implementation, created greater speed and efficiency in the information system for lab results. Although the deployment of this information system has led to a speedier dissemination of the results to physicians, most of these professionals do not use this system in the laboratory facilities, lacking familiarity with the internet and computer use. There was only one exception, among the family doctors linked to the facility who regularly accessed the GAL: a team composed of younger physicians.

Additionally, not all health facilities in the public system have the necessary equipment to access the GAL system (computers and/or internet). Some still need to contact the lab secretary to get test results.

Still regarding the information system, we identified several systems present in the unit, besides GAL: a virtual internal parallel information system, patient record files (on paper), information from community health workers on patients’ treatments, stored in shoeboxes, and the lab technicians’ record notebook, with notifications of samples and test results.

The main issue that stood out in the rollout of the new technology at the Manaus facility was the change in the workflow within the laboratory. With the smear microscopy, the work process involved the preparation and reading of two samples per user. The process of preparation and reading a single slide lasts around 30 min. Workers reported that the job was a routine that involved contact with an abrasive substance, foul odors, and poor ergonomic conditions, as they spent their shifts hunched over microscopes. Conversely, with the new technology, technicians prepare the samples for inserting them in the instrument. The preparation time is about 15 min. At the facilities participating in this study, each instrument works simultaneously with four samples. At the site in Manaus there are two instruments in operation. As the instruments do their processing, the laboratory technicians are dedicated to other activities, such as smear control and HIV testing. This can be seen in the exchange below, from the Manaus session (P is a physician, T a lab technician, R1 is one of the field researchers):

P: Look, the routine is like this. 15 min, you wait, put in the machine. Then, when it strikes 1 h and 45, the lab people returns about 15 min before reading the result to begin preparing the second batch of samples.

T: Yeah, we do not waste time.

P: You can not prepare it much earlier, has to be 15 min. Then you have to wait 15 min. Then, when that sample is finished, take one and put the other. Then, you wait a further 1 h and 45 to start preparing other samples.

L: Yeah, that’s how we do it.

P: In these intervals, they will do the control smears, have a snack.

R1: They get time to do other things. So, the additional time they get, they get ahead with the control smears… got it.”

Finally, the lab workers reported that the implementation of the new technology has had a very positive impact on the improvement of working conditions in the laboratory. They do not have to constantly deal with fire, nor with foul odors. The ergonomic conditions improved considerably since the technicians do not need to spend their day poring over microscopes. Another fragment from Manaus illustrates this (P is a physician, R1 one of the field researchers):

“P: And for the smear, they used to spend the whole morning there burning that thing, that awful smell, and all afternoon on a microscope with a heck of a back pain, take out a slide, put another slide in…

R1: So, look, when a technician puts the samples on the machine, we have to make an observation: there is spare time to do the control smear and… [person] is talking about something important because in addition to having spare to make the control smear and then, balancing the process, you have better working conditions, because of the things that wear down the worker, like the smell… better working conditions, let’s put it this way.

P: Because there, they spend a whole morning with that dye, that awful fire, that bad smell…

R1: There’s temperature, smell, ergonomic performance…

M: And the whole afternoon reading slides with no rest. Take out one, put another in, right? 3 microscopes, 3 persons reading slides with no interruptions…

M: Sitting 4 h, on a microscope, reading slides, it is a heck of a back pain.”

As in Rio de Janeiro, a member of the administrative staff was assigned the task of entering data in the GAL.

### The managers’ perspective

The codes used in the content analysis of the interviews were as follows:

- pros: the perceived advantages of the new technology;
- cons: the perceived problems with the new technology;
- changes: what were the major changes detected by the managers;
- sustainability: how they evaluated the perspectives of widespread and continued use of the new technology in the public health care system.

Managers interviewed considered the technology itself mostly in positive terms, but expressed concerns related to sustainability and the rollout of the information system to ensure its benefits. Among the positive points mentioned, the addition of rifampicin resistance detection, the accuracy and reliability of the results, the less burdensome processes for lab workers and shorter time to an accurate results were consistently highlighted. Negative aspects or reasons for concern were mostly related to the efficiency of the health system and its ability to make the most of the new technology considering the need for better data management, ensure adequate maintenance and sustainability and full rollout of the GAL system. The general perception was that, despite the changes required, the workflow and the management of samples between labs and care facilities were not significantly disrupted and adaptations to the new method were swift.

#### The pros of the new technology

The managers pointed out several aspects in favor of the new technology. The ability to identify resistance to rifampicin was unanimously praised by respondents:

“It is a more qualified result, we can identify rifampicin resistance in that patient. This also means that tuberculosis patients’ care will be quicker and more qualified by the health care staff.” (Manager 2, Rio).

“The diagnosis also informs you on the issue of resistance to rifampicin. Upon learning that the patient is already resistant to rifampin, this helps with the issue of patient severity, because the orientation of the Ministry of Health is that, if it’s resistant, we’ll wait for the old culture method, all that…” (Manager 3, Manaus)

Another issue highlighted was the reliability of the technology, providing the most accurate diagnoses:

“Its reliability is total. It’s out of this world! It is something magical! In the period from August 3rd to 25th, we performed 304 exams with the ‘Gene’ and 34 tested positive. Of these 34 positive samples, all were tested by smear, around six slides were negative. That is, these patients would pass right through and would continue to spread the disease.” (Manager 3, Rio)

“The test has a higher sensitivity, higher specificity, so we better qualify the diagnosis of tuberculosis.” (Manager 2, Manaus)

One respondent pointed out the importance of the new technology in the control of the workflow in the laboratory:

“The machine also has its importance from the point of view of monitoring, auditing what happens inside the lab. With smears, you did not know what was going on. I mean in quality, errors if the technique is being done properly.” (Manager 1, Rio)

The satisfaction of the professionals working in the area was cited by all respondents in Manaus:

“This new methodology was an incentive for professionals already working in the area, which for many years did not see any innovations in tuberculosis. Then, the emergence of this new methodology gave a new boost to many people.” (Manager 1, Manaus)

“The satisfaction of employees as well, because for them it was so … a very good thing for them. Just not having to do 100 smears per day, 100 cultures.” (Manager 3, Manaus)

One respondent also cited the benefits to worker’s health:

“The emotional and psychological factors of the workers who will be most pleased to do its work, will get sick less often, take fewer licenses, will be less prone to giving up working in that area. We saw a great satisfaction.” (Manager 1, Manaus)

Biosafety issues and worker’s protection were also mentioned:

“I think the main advantage [of the Xpert MTB/Rif] refers to biosafety, the minimal structure that is needed to perform the procedure with the equipment. Just a workbench with plug, and it is sufficient to install the equipment and do the exam. Unlike smears that require a necessary structure, adequated to the issues of biosafety, ventilation, a more adequate area. (…) The worker protection, which is sometimes required to work in a hazardous space, which does not have the proper conditions, and even sometimes does not receive all the necessary protective gear for biosafety. This is very common, especially in the countryside, where we see pathology technicians doing sample preparation without masks in any sink, in a hallway.” (Manager 2, Manaus)

Another issue highlighted was the reduction in required resources:

“If I performed 100 tests per day, I spent with the previous method 200 slides because it requires two samples. I needed to have dyes, slides, all doubled. The material was doubled. Because I had to make two samples (…) With the implementation of the ‘Gene’, no, not only one sample was worked on. Then all the required resources are reduced. If you do a test you will not use anymore those dyes, all those things. Why? The diagnosis is made directly on the machine.” (Manager 3, Manaus)

Reducing the time of diagnosis was identified as an advantage of the new technology by respondents:

“With the implementation of the ‘Gene’ only one sample became [necessary]. Then, the next day the patient had the result, or we told the patient to wait, he came to give us [the sample] in the morning and in the afternoon he could check with us the result that was already delivered. Before we did [the diagnosis] in three days, 72 h. Why? Collect the first sample, collect the second and 72 h have passed.” (Manager 3, Manaus)

Respondents also emphasized the ease of training the new technology:

“With smears, training is much more complicated, you need a lab bench, you need a microscope, the schedule for training is much more complex. And with the automated equipment it would be an automated thing and I believe training is very simple, just operate the equipment.” (Manager 2, Manaus)

#### The cons of the new technology

Most of the interviewed managers had difficulties in finding disadvantages in the new technology. Sentences like “I see nothing against it” or “I only see pros, no cons” were common:

“I personally can’t see any downside. Compared to smears, I see nothing that I could say: 'Ah! It would be better to stick to smears. I can’t see that, particularly.” (Manager 2, Manaus)

However, some respondents mentioned the difficulty of professionals at the front lines of health care to interpret the results produced by the new technique:

“When you have a positive result from the ‘Gene’ and a negative result from smear and do not qualify the intensity with [a number of] crosses, people [health care professionals] keep calling, they are in doubt. They are trying to quantify the positive result, they are used to it. Now there is no more quantification, then they get concerned, thinking that the result is wrong. We have to stop and explain. I mean, the lab loses time explaining to people how the test was done and why it was not quantified.” (Manager 5, Rio)

One respondent highlighted the challenge of getting the necessary funding for maintaning the new technology:

“The challenge is to get money to buy cartridges and maintain what has been started. While there is still resources left from this project it is assured, but after that, which is unfortunate, is that we go back to the same situation before, especially in facility X, which has the highest demand of our state. There, the problem is greater than anywhere. The downside is this: how to get resources to ensure that it is kept in the same proportion that was made during the implementation of the project.” (Manager 1, Manaus)

#### The information system

Respondents in Rio de Janeiro were unanimous in saying that the information system (GAL) presents itself as a challenge, with implications for the agility of the results produced by the new technology:

“We are in a phase of implementation of the GAL, which is a Health Ministry system that allows web access to all results, and this is not fully consolidated as we would like. So the agility we expected with the ‘Gene’, I think in practice, we still need to improve the use of the GAL system so that we actually get this result.” (Manager 6, Rio)

Some managers mentioned that GAL lacks infrastructure to operate with full functionality:

“The problem of the system is that the [healthcare] network is not completely structured in a way to work online. So not all units achieve a good connectivity. Do all of them have a computer? They all have. The policlinic, for example, has computers, but I do not have a computer in my office. I have no way to enter a test requisition in my sample collection room. Then, requisitions have to follow a flow in order to send this request somewhere where there are computers, where there’s good connectivity, so that we can enter the data.” (Manager 5, Rio)

Another issue that arose was the fact that when the facilities are not capable of making a test request through the system, they do not have access to the results through the GAL:

“Not all units have the same structure. So, what happens, we often receive samples in the laboratory with the requisition on paper and not in the system. Then, the laboratory has to enter the requisition and the result. And often, the facility can’t see the result, either. The GAL, I find it excellent, the problem is having the structure in order to harness the full potential of the system.” (Manager 5, Rio)

Despite the difficulties of infrastructure for the operation of the GAL system, respondents stressed that it is an important tool for the viability of new diagnostic technologies:

“We knew that if we did not couple it [the new diagnostic technology] with an information system that made the test results flow, we would be doing a compromised assessment. So, we anticipated some issues. So we are not seeing, for example, as if a Rolls Royce, which has the potential to run at 200 km/h, but won’t be able to do so on a bumpy road. We discussed and anticipated it, minimized this in the deployment itself. And that way we did not create an artificial thing, because the GAL is an Internet-based system and what we did was to accelerate its deployment so as to be able to address the issue of implementing a new technology in an environment that would not compromise our ability to evaluate.” (Manager 1, Rio)

#### Changes in workflow

The new diagnostic technology resulted in a change in the work process in the laboratory:

“The machine is considered a quick test, but it takes actually two hours. The machines that we are working with have four slots, then in fact the technician can only put four tests to cycle every two hours. So it ends up being a modifier in the work process in the laboratory, since before, with smears, the technician could arrive in the morning, stain all the slides and read them throughout the day, and whoever stained was not necessarily the person who would read.” (Manager 1, Rio)

This manager clarifies that changes in work processes are related to actions laboratory, not interfering in the front lines:

“The technology is not disrupting work processes at the front lines, that would be in fact more complicated. That is, I’m taking samples in the same way, I am forwarding samples in the same way, I’m waiting for and getting samples within a system that was already expected to receive, considering that the GAL was about to be deployed despite the Xpert project.” (Manager 1, Rio)

“It was a new thing and it messed a little with the structure of the laboratory, in the sense that people needed training, and it was something that took quite a while given the very activities that the ‘Gene’ required at that moment. And at that initial moment, I had to suspend the routine work in the laboratory. Routine tests, clinical analysis. So there was an exclusive dedication to the ‘Gene’ due to our demand here, which is immense.” (Manager 3, Manaus)

Respondents indicated that the new technology generated a decrease in the work load of the laboratory:

“The other advantage is to reduce the load of work in the laboratory. From the moment in which we work with only one sample, we can organize ourselves better for case detection. (…) The main change was in the lab, the operationalization of the test, and improving the workflow in the laboratory we can further intensify this surveillance, because the lab will respond to it. So when I had the smears, we limited ourselves: ‘No, there is a quantity limit.’ Then we restrict ourselves from making a more intense search [for cases]. With the change of the lab, we have a freer lab for us to have a larger number of persons to be examined.” (Manager 2, Manaus)

In relation to changes in workflow, no resistance or difficulties were identified in the interviews:

“There was no difficulty. It was very easy. People were trained. It was very smooth.” (Manager 3, Rio)

One manager mentioned the good adaptation of the technicians to the new technology:

“The machine software is in English, which is a limitation, but as everything is very standardized, they [technicians] have adapted very well.” (Manager 1, Rio)

Another interviewee even reported a fascination produced by the machine:

“All technicians who are working there are enchanted!” (Manager 3, Rio)

One respondent stated that technician satisfaction was related to the fact that their routine work became more similar to other laboratories working with computerized systems:

“From what I saw of the satisfaction of technicians who are already using it, it brings the routine of the tuberculosis lab closer to the mode of operation of other laboratory areas, which are automated and all work already with computerized systems. Tuberculosis was still somewhat apart, because it was the one that had to perform a very old technique. So the technicians were very happy to work with it.” (Manager 1, Rio)

#### Perspectives for the deployment of the new technology in the public healthcare system (SUS)

The interviewees were unanimous in that the deployment of new technology in the SUS would bring benefits for the diagnosis and treatment of TB:

“Our hypothesis is that the number of cases that are going to be treated with definitive diagnosis will be greater. Today we have around 50 % of patients who start treating tuberculosis with a correct diagnosis. We hope to increase this number which is certainly a quality indicator for the Health Programme.” (Manager 3, Rio)

“We will detect most cases, we will more adequately, and consequently, we can avoid more deaths than we have been able to do.” (Manager 1, Manaus)

“I believe it will be a breakthrough in the treatment of tuberculosis, which is a disease that primarily affects our county, Rio de Janeiro. So I believe that the city has everything to gain with this new method, in advance of multidrug resistance, resistance in tuberculosis. Already having a diagnosis where the patient is detected if it is resistant to some drugs or not, his treatment is completely changed.” (Manager 2, Rio)

Respondents showed, however, concerns regarding the necessary allocation of financial resources for the maintenance of the new technology:

“Who will maintain [the new technology]? From where will come the resources to keep it running? So, this will depend a lot on pacts between the Federal Government, states and municipalities. Maybe the Ministry [of Health] will contribute with the equipment and the municipalities will bear the costs of the kits… I think it will depend on how this conversation will be negotiated. I think the challenge will be in the pacts, in defining this, because when it comes to resources everybody tightens the strings of the purse.” (Manager 2, Manaus)

### Limitations of the study

The main limitation of the study is its context specificity; the data collection was made in specific settings of two cities in Brazil, and any conclusions and inferences are limited by this. The selection of the sites, however, was done in accordance to the high prevalence of TB, on the one hand, and the different stages of implementation of the new technology, which confers a degree of representativeness with regards to the situation of the Brazilian healthcare system. Managers and professionals were purposefully selected in accordance to their relevance in the sites, and can be considered representative in that sense as well. Patients were selected by convenience, but the utilization of the saturation criterion gives us confidence that their views are also representative of the users of public healthcare in the country.

Having said this, however, we believe that the lessons learned from this specific process of implementation of a new technology can at least inform researchers and managers operating in similar contexts in understanding the complex factors that influence the acceptance of newly introduced technology.

## Discussion

The acceptance and dissemination of a new technology in health care is a non-linear process, that can be determined by a large array of factors, some linked to the technology itself or the associated health problem, others related to actions and interests of the different stakeholders [10, 19]. Scientific evidence of a potential superiority with regard to methods already in use is but an element to influence the adoption of a new technology. The capacity of organizations to incorporate new knowledge and practice, the institutional, social and political forces involved, and the perceptions and reasoning of the various actors participating in the multiple arenas where the incorporation of a new technology takes place may induce specific patterns of acceptance or rejection that need to be understood at a local level [20].

The incorporation of the new TB diagnostic technology discussed in this study seems to be taking place without much difficulty, but there are some limiting factors to fully taking advantage of its benefits. The difficulties with the online system echo observations made in other studies about problems with health information systems in general [21]. The low diffusion of the knowledge required for effective use of equipment and networks, the low availability of this resource type in public and the very proliferation of systems that do not interact with each other, often requiring the reinsertion of data that should already be available, lead to bottlenecks in the flow of information, which in our case even reflected in the selection of patients for interview.

Selecting patients who had been diagnosed by the new method demanded additional time, precisely because of the difficulties still existing among health care workers regarding access to the GAL. We view with concern the adopted solution, both in Rio and Manaus, specifically to designate an administrative staff member to enter data into the system, repeating a tradition of restricting the user interface of the system to a few administrative professionals, when by design it should be accessible to everyone involved.

This bottleneck can undermine one of the great advantages of the system, which is its speed. We observed that the practice of starting treatment with only the clinical diagnosis still persists; unless laboratory confirmation is available as soon as possible, this is unlikely to change. Nevertheless, we did witness a few examples of the desired change in clinical behavior. In interviews with managers in Rio de Janeiro, a circumscribed problem also emerged, in that certain doctors experienced difficulty to interpret the results produced by the new technology. It should be noted that, in general, even with quick access to test results, changes in medical conduct depend on a significant investment in the continued education of these professionals, which should perhaps be considered more carefully in the routine rollout of the new system.

A particular problem in the case of Manaus is the long time it takes patients to get to the testing facility, given the local practice of referring patients to a centralized facility. Although this is an aspect linked to the specific organization of public health services at that location, not the testing technology *per se*, it is a factor that may ultimately adversely impact the access to the test. Both in Manaus and in Rio, displacement is facilitated by the fact that patients are largely on medical leave, a contingency that must also be considered in designing the overall strategies to diagnose and treat TB in the general population.

From the patients’ perspective, changes seem less noticeable. The concerns that we have identified are linked to the disease itself and its stigmas, and unlikely to be impacted by any diagnostic technology.

The acceptance by managers was also wide and practically unrestricted, the only relevant question that arose in the interviews was about the sustainability of long-term use of the new technology. Given the uncertainties that unfortunately persist with regards to SUS funding, this is a relevant issue, but not specific to the adoption of any given technology.

A particularly encouraging finding is the virtually unrestricted acceptance of new technology by lab technicians. Resistance at this level could jeopardize the adoption of the new process, and we could not observe any indication in that direction. From this particular case, a general recommendation for the implementation of new technologies could be derived: the participation and empowerment of professionals directly involved is critical. We can also speculate on the effect of positive symbolic adoption of a technology in a field that has spent decades without major innovations.

Other than that, although there are attempts in the literature to systematize factors that can facilitate or hinder the adoption of new technologies [11–13, 22–24], it seems more likely that the specific reasons in each case are more contingent than algorithmically predictable [25]. Rogers [26] describes some of the attributes of an innovation that can favor its successful incorporation: comparative advantages with regard to preexisting technologies; reduced complexity; compatibility with values, needs and current practices within organizations; opportunities for experimentation offered by the new technology; visibility of results of the new technology to its users. Other authors emphasize evidence as a factor that works in favor of the acceptance of the new technology, but what constitutes “evidence” can vary across different groups [27]. In this particular case, pragmatic and cultural factors played a significant role, namely, making work in the laboratory easier but still within control of the technicians, and the relative fascination with “new” things. Kulviwat et al.’s suggestion that self-efficacy also plays a role in technology acceptance [28] may apply here as well, at least in the case of the lab technicians. Available evidence of the intrinsic qualities of the new technology, on the other hand, was not enough to make physicians change their routine of starting treatment before laboratory confirmation.

## Conclusion

In summary, the introduction of the new technology has been widely accepted and is viewed very positively by those involved, especially laboratory technicians. The process of adoption itself was also uneventful and did not face major barriers by managers or health professionals. Most of the difficulties encountered are more attributable to contextual conditions than to the technology itself, but must in any case be considered for its expansion throughout the national SUS network.
